# ﻿A Taxonomic Odyssey: An annotated checklist of *Peromyscus* (Cricetidae, Rodentia) in Honduras

**DOI:** 10.3897/zookeys.1218.126535

**Published:** 2024-11-22

**Authors:** Celeste M. López, Manfredo A. Turcios-Casco, Eric van den Berghe, Nicté Ordóñez-Garza, Martin R. Alvarez

**Affiliations:** 1 Programa de Pós-Graduação em Zoologia, Departamento de Ciências Biológicas, Universidade Estadual de Santa Cruz, Ilhéus, BA, Brazil; 2 Asociación para la Sostenibilidad e Investigación Científica en Honduras (ASICH), Francisco Morazán, Honduras; 3 Centro Zamorano de Biodiversidad, apartado 93, Ambiente y Desarrollo, Escuela Agrícola Panamericana, Francisco Morazán, Honduras; 4 Department of Ecology and Evolutionary Biology, University of Michigan, Ann Arbor, USA; 5 Michigan Pathogen Biorepository, University of Michigan, Ann Arbor, USA

**Keywords:** Central America, Deer mice, geographical distribution, historical review, systematics, taxonomy

## Abstract

Deer mice, *Peromyscus*, thrive in diverse environments and altitudes across North and Central America. The number of extant species continues to be debated with species counts ranging from 53 to 83. This study represents the first comprehensive historical and taxonomic account of the genus *Peromyscus* for Honduras. We systematically compiled records from all available sources, incorporating verified genetic and morphological evidence. We confirm the presence of *P.beatae*, *P.cordillerae*, *P.nicaraguae*, *P.salvadorensis* and *P.stirtoni* for Honduras. The distribution maps provided here include confirmed records and approximate localities in a few cases and offer insights into the geographical distribution of these species in Honduras. Conducting a comprehensive assessment of the taxonomic status of *Peromyscus* in Honduras is imperative to achieve accurate conservation assessments within the larger Mesoamerican landscape. The present review establishes the baseline for future research on deer mice in Honduras, aiding in the validation of distributions and ecological data for the poorly understood genus *Peromyscus* in the country.

## ﻿Introduction

Deer mice, family Cricetidae Fischer, 1817, subfamily Neotominae Merriam, 1894 (Pardiñas et al. 2017), genus *Peromyscus* Gloger, 1841 defined by Platt et al. (2015), are a diverse group. According to Dawson (2005), deer mice underwent significant diversification during the Pleistocene, and they are currently distributed from Alaska to Panamá (Hall 1981; Bradley et al. 2016; Pérez-Consuegra and Vázquez-Domínguez 2016). *Peromyscus* thrive in diverse habitats, encompassing deserts and rain forests in both temperate and tropical climates (Tiemann-Boege et al. 2000) from sea level to 4300 meters above sea level (Bedford and Hoekstra 2015).

The high rate of diversification has posed a challenge in clarifying the taxonomic relationships within this genus and has generated ongoing controversy regarding the number of *Peromyscus* species (Osgood 1909; Carleton 1989; Bradley et al. 2007; Miller and Engstrom 2008; Pérez-Consuegra and Vázquez-Domínguez 2015). Hooper and Musser (1964) initially proposed 59 species within the genus *Peromyscus*. Subsequently, Hooper (1968) reduced the count to 57 species, upheld by Carleton (1989) and Musser and Carleton (1993). However, Musser and Carleton (2005) reduced the count to 56 species. Later, Platt et al. (2015), confirmed 53 species based on genetic analysis. Pardiñas et al. (2017) and Hernández-Canchola et al. (2022) then suggested 66 species. Presently, the Integrated Taxonomic Information System–ITIS (2024) recognizes 58 *Peromyscus* species, in contrast with the 83 species cataloged by the American Society of Mammalogists in their Mammal Diversity Database (ASM 2024).

In the last ten years, we have witnessed a significant shift in our understanding of deer mice taxonomy and systematics based on a series of prominent research studies (e.g., Pérez-Consuegra and Vázquez-Domínguez 2015, 2016; Platt et al. 2015; Bradley et al. 2016, 2017; Álvarez-Castañeda et al. 2019; Kilpatrick et al. 2021; Bradley et al. 2022). These studies have been pivotal in understanding the systematic, taxonomic, and biogeographical diversity within *Peromyscus* in the Mesoamerican region.

In Central America, approximately 15 species of deer mice have been documented (Musser 1969; Bradley et al. 2000; Ordóñez-Garza et al. 2010; Trujano-Álvarez and Álvarez-Castañeda, 2010; Lorenzo et al. 2016; Matson et al. 2016; Pérez-Consuegra and Vázquez-Domínguez 2016; Álvarez-Castañeda et al. 2019; Ramírez-Fernández et al. 2023; ASM 2024). However, in most Central American countries, the total number of species has not yet been conclusively determined. This is due to the synonymy of some species (e.g., Kilpatrick et al. 2021). while some subspecies have been elevated to species level (e.g., Pérez-Consuegra and Vázquez-Domínguez 2015). This shifting landscape highlights the taxonomic complexity within this genus and underscores the need for ongoing research to achieve a more precise understanding of the number of species in northern Central America, including Honduras. For example, 13 species are recognized in Guatemala (five endemic), four in El Salvador, and three in Nicaragua (ASM 2024). We explored the latest scientific literature and historical revisions of *Peromyscus*, specifically focusing on specimens from Honduras to construct a comprehensive annotated checklist of this genus in Honduras. We relied on specimens housed in museums or that were confirmed by genetic and morphological studies to generate distribution maps.

## ﻿Materials and methods

### ﻿Study area

Honduras covers 112,492 km^2^ making it the second-largest country in the Central American Isthmus (Hernández Oré et al. 2016). Positioned at the core of Central America, this region is one of the Earth’s biodiversity hotspots (Mittermeier et al. 1999), owing to its elevation and climatic diversity.

**Figure 1.**
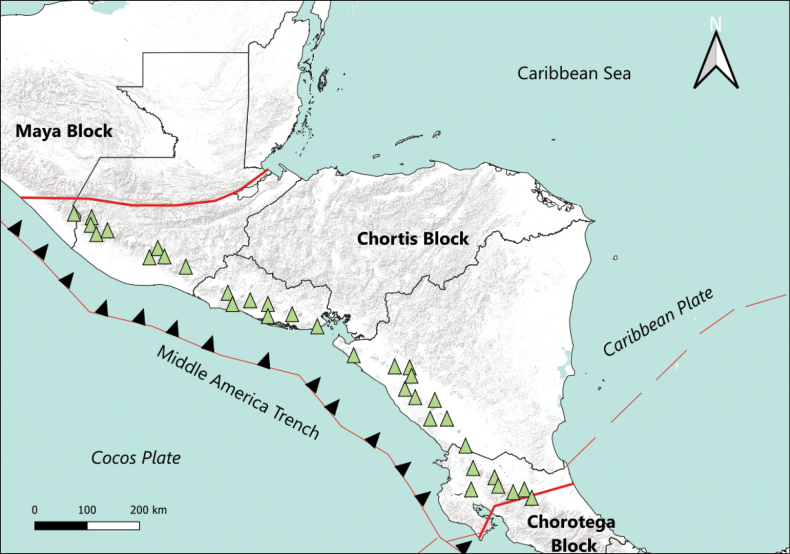
Representation of the Chortís Block within Central America. Adapted from Marshall (2007).

Geologically, the Honduran territory is part of the Chortís Block (Fig. 1), which includes the western highlands and the central plateau of Chortís (Marshall 2007) in the Isthmus of Tehuantepec (Dengo 1968). This area is characterized by mountain ranges separated by a discontinuous series of north-trending small rift valleys, featuring late Miocene to Quaternary soils (Burkart and Self 1985; Marshall 2007). The mountainous geography results in notable elevations in various parts of the country with notable peaks that include 2870 m a.s.l. in Celaque National Park to the west, 2454 m a.s.l. in the Nombre de Dios mountain range, and 2435 m a.s.l. in Pico Bonito National Park to the north. In the northwest, in the Santa Barbara Mountain National Park elevations reach 2777 m a.s.l., whereas in the central region they reach 2420 m a.s.l., and in the eastern region, they attain 2351 m a.s.l. (Townsend 2014; Matson et al. 2016). This mountainous topography gradually gives way to the Lowland Province of the Mosquitia Coast (<450 m a.s.l.), characterized by an extensive alluvial plain in the eastern strip of the Caribbean coast (Marshall 2007). The Honduran Pacific coast, featuring the Gulf of Fonseca, is characterized by extensive estuaries, lagoon systems, and mangrove forest in coastal plains, punctuated by volcanoes (Bengtson 1926; Dunbar et al. 2020).

### ﻿Preserved specimens

We compiled historical records of *Peromyscus* in Honduras using Goodwin (1941, 1942) as a basis, as well as information available on Global Biodiversity Information Facility–GBIF.org (2023) concerning the specimens housed in the museums. This allowed us to gather a broad range of taxonomic and distributional information on *Peromyscus* in Honduras. We also reviewed the modifications over time in the taxonomic classification of the species of *Peromyscus* living in Honduras (e.g., Hall and Kelson 1959; Musser 1969; Carleton 1989; Bradley et al. 2000, 2007; Ordóñez-Garza et al. 2010; Trujano-Álvarez and Álvarez-Castañeda 2010; Pérez-Consuegra and Vázquez-Domínguez 2015, 2016; Bradley et al. 2016; Matson et al. 2016; Kilpatrick et al. 2021; Bradley et al. 2022; León-Tapia et al. 2022).

### ﻿Taxonomic accounts

We considered only those species, whose identification has been confirmed by external and cranial morphology and morphometry (Goodwin 1942; Musser 1969; Carleton 1979; Matson et al. 2016). We also reviewed the literature (Hall 1981; Musser and Carleton 2005; Trujano-Álvarez and Álvarez-Castañeda 2010), karyotypic analyses (Bradley and Ensink 1987; Peppers et al. 1999), cytological-taxonomic studies (von Lehmann and Schaefer 1979), as well as biogeography and phylogenetic studies (Sullivan et al. 1997; Bradley et al. 2000; Bradley et al. 2007; Pérez-Consuegra and Vázquez-Domínguez 2015, 2016; Bradley et al. 2016; Kilpatrick et al. 2021). This information is summarized in Tables 1–3.

**Table 1.** Summary of the systematic history and taxonomical arrangements of *P.beatae* in Honduras.

Reference	Taxonomical history of *P.beatae*	Scope of their methodology
**Goodwin (1942)**	*P.boyliisacarensis* was the only one of *P.boylii* group	External and cranial morphology and morphometry of collected specimens
**Hall (1981)**	*P.b.sacarensis* was maintained as a subspecies	Based on external and cranial descriptions of museum specimens and marginal records of the distribution
**Bradley and Ensink (1987)**	Continued to recognize the subspecies *P.b.sacarensis*	Karyotypic analyses
**Bradley et al. (2000)**	Reassigned *P.b.sacarensis* with *P.beatae*	Analysis of the mitochondrial cytochrome *b* gene in *P.b.sacarensis*
**This study**	Recognized *P.beatae* as the only species of the *P.boylii* group in Honduras	Bibliographic taxonomic review

**Table 2.** Summary of the systematic history and taxonomical arrangements of *P.cordillerae* in Honduras.

Reference	Taxonomical history of *P.cordillerae*	Scope of their methodology
**Goodwin (1942)**	Considered *P.mexicanussaxatilis* and *P.hondurensis* as separate species	External and cranial morphology and morphometry of collected specimens
**Musser (1969)**	Referred to the specimens previously cited as *P.mexicanussaxatilis* and *P.hondurensis* to be *P.oaxacensis*	External and cranial morphology and morphometry of preserved specimens
**von Lehmann and Schaefer (1979)**	*P.hondurensis* was still considered a valid species even though it had already been synonymized by previous studies	Cytological-taxonomic studies, including sperm analysis, morphology, and comparative cytochemistry
**Carleton (1979)**	*P.aztecusoaxacensis* was synonymized with *P.oaxacensis* and *P.hondurensis*	External and cranial morphology and morphometry of preserved specimens.
**Hall (1981)**	Referred to specimens previously cited as *P.oaxacensis* and *P.hondurensis* to be *P.oaxacensis*	Based on external and cranial descriptions of museum specimens and marginal records of the distribution
**Sullivan et al. (1997)**	Presented evidence indicating that subspecies *P.a.oaxacensis* in the south and east of the Isthmus of Tehuantepec represents a distinct species	Phylogeography based on phylogenetic analyses of 668 bp of the mitochondrial cytochrome *b* gene
**Musser and Carleton (2005**)	Continued to recognize the subspecies *P.a.oaxacensis*, emphasizing the need for further scrutiny in the populations mentioned by Sullivan et al. (1997)	Bibliographical review
**Matson et al. (2016)**	Considered *P.oaxacensis* as the species for Honduras within the *P.aztecus* group*	External and cranial morphology and morphometry of collected specimens
**Kilpatrick et al. (2021**)	Recognized the subspecies *P.cordilleraehondurensis* for the population in Honduras considered as *P.a.oaxacensis*	Molecular data from the mitochondrial cytochrome *b* gene
**This study**	Consider *P.cordilleraehondurensis* for all representatives of the *Peromyscusaztecus* group for Honduras including the following synonyms: *P.oaxacensis*, *P.hondurensis*, *P.aztecus*, and *P.a.oaxacensis*	Bibliographic taxonomic review

*Matson et al. (2016) relied on the description by Bradley et al. (2014) for their records in eastern Honduras. However, Bradley et al. (2014) did not utilize specimens from the *P.aztecus* group from Honduras in their comparisons.

**Table 3.** Summary of the systematic history and taxonomical arrangements of *P.nicaraguae* and *P.salvadorensis* in Honduras.

Reference	Summarized taxonomical history of *P.nicaraguae* and *P.salvadorensis*	Scope of their methodology
**Goodwin (1942)**	Considered *P.mexicanussaxatilis* and *P.guatemalensistropicalis* as separate species	External and cranial morphology and morphometry of collected specimens
**Musser (1969)**	Referred to specimens cited as of *P.guatemalensistropicalis* to be *P.m.saxatilis*	External and cranial morphology and morphometry of preserved specimens
**Hall (1981)**	Maintained *P.m.saxatilis* as the species to occur in Honduras	Based on external and cranial descriptions of museum specimens and marginal records of the distribution
**Bradley and Ensink (1987)**	Supported the recognition of *P.m.saxatilis* for Honduras	Karyotypic analyses
**Trujano-Álvarez and Álvarez-Castañeda (2010)**	Considered *P.m.saxatilis* to occur in Honduras	Mammalian Species review for *P.mexicanus*
**Pérez-Consuegra and Vázquez-Domínguez (2015, 2016**)	Resurrected *P.nicaraguae* and *P.salvadorensis* from synonymy with *P.m.saxatilis*	Molecular analyses of the mitochondrial cytochrome *b* gene, phylogenetic studies, and assessments of genetic diversity and lineage differentiation
**Bradley et al. (2016)**	Reaffirmed *P.nicaraguae* as a valid species in Honduras.	Mitochondrial DNA analysis of the cytochrome *b* gene
**Matson et al. (2016)**	Supported the designation of *P.nicaraguae* and *P.salvadorensis* as proper species that occurs in Honduras	External and cranial morphology and morphometry of collected specimens
**This study**	Recognized *P.nicaraguae* as well as two morphotypes of *P.salvadorensis* supporting the hypothesis of Pérez-Consuegra et al. (2015, 2016)	Bibliographic taxonomic review

**Figure 2.**
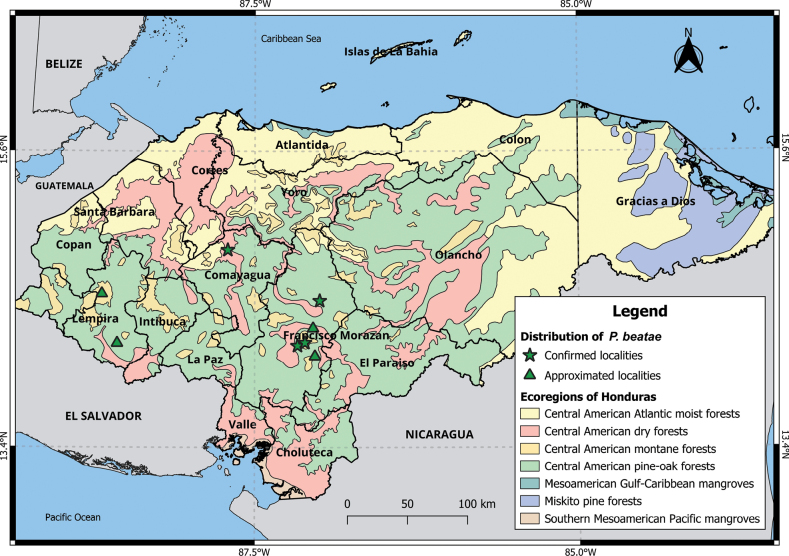
Distribution of *P.beatae* in Honduras.

**Figure 3.**
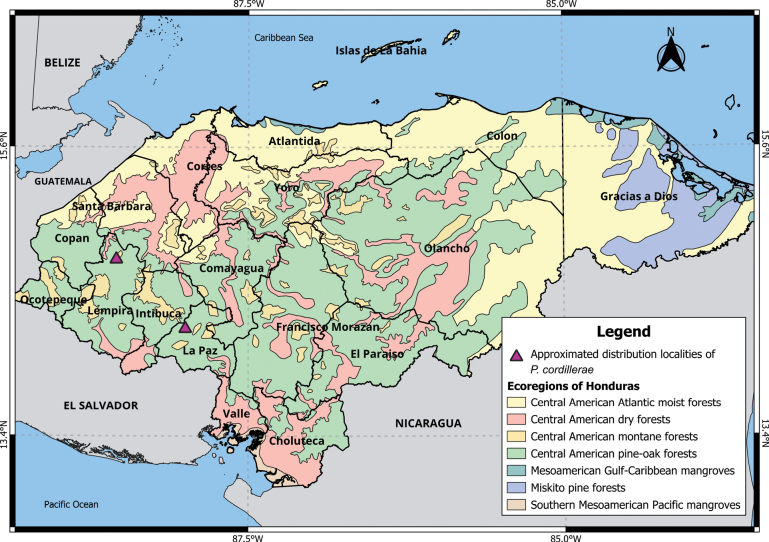
Distribution of *P.cordillerae* in Honduras.

**Figure 4.**
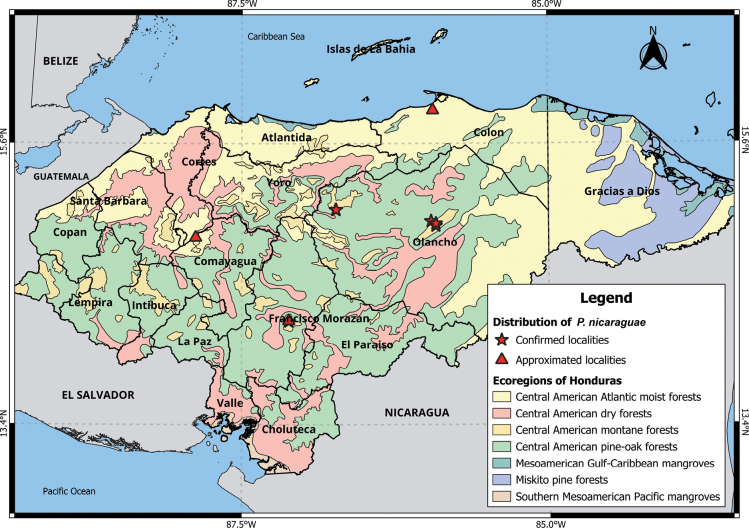
Distribution of *P.nicaraguae* in Honduras.

**Figure 5.**
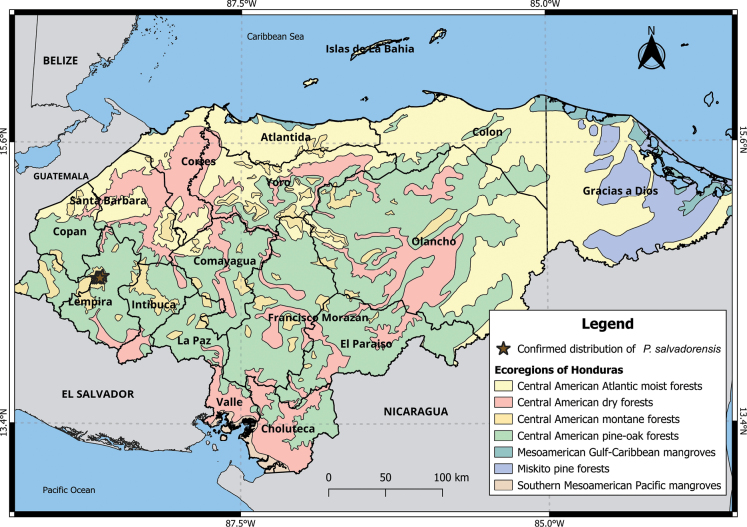
Distribution of *P.salvadorensis* in Honduras.

**Figure 6.**
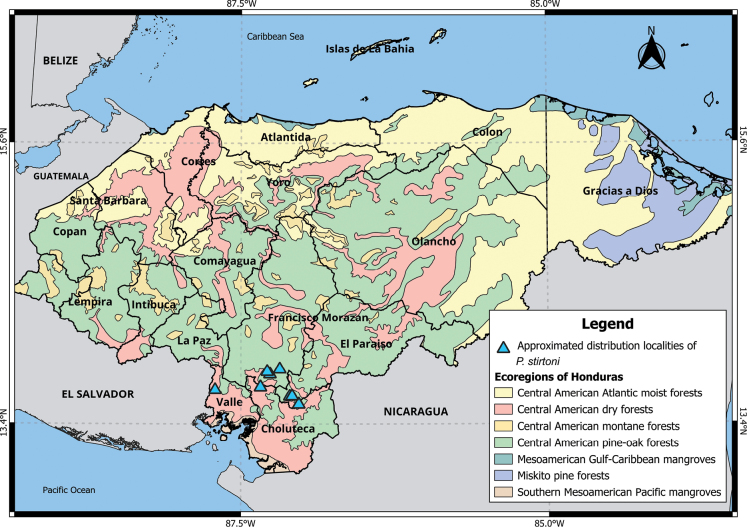
Distribution of *P.stirtoni* in Honduras.

The GBIF.org dataset (2023) of museum specimens was downloaded for the distributions of *P.beatae* Thomas, 1903 and *P.stirtoni* Dickey, 1928 because these are the only species that had not experienced substantial taxonomic changes; and for *P.stirtoni* we did not present any table because its epithet has not changed since its description (Jones 1990). Citizen science observations (e.g., iNaturalist) were not considered for any species; additionally, we present the elevation ranges as well as the departments and the ecoregions, where their occurrence has been confirmed (see Figs 2–6; Suppl. material 1).

### ﻿Distribution map

For the creation of the distribution maps, we used QGIS Desktop software version 3.28.11 and included only the records that were confirmed following the previously mentioned criteria. Specimens without locality coordinates were approximated based on the verbatim descriptions (see Figs 2–6; Suppl. material 1). Elevations were also corroborated (see observations in Suppl. material 1). The ecoregions defined by Olson et al. (2001), were presented for each species (see Figs 2–6; Suppl. material 1).

### ﻿Museum abbreviations

The museum abbreviations are as follows: **AMNH** = American Museum of Natural History; **CMNH** = Carnegie Museum of Natural History; **TCWC** = Biodiversity Research and Teaching Collections, Texas A&M University; **TTU** = Texas Tech University Museum; **UF** = Florida Museum of Natural History; **USNM** = Smithsonian Institution, National Museum of Natural History; and **UNAH** = Universidad Nacional Autónoma de Honduras.

## ﻿Results

Based on our examination it seems that five species are present in the territory of Honduras, and 825 specimens of *Peromyscus* are housed in the zoological collections in natural history museums; from the latter, we only considered 254 specimens that were confirmed by recent studies and in accordance with the current taxonomy (see Suppl. material 1). About a third of these museum specimens have been employed in studies to report on this group within the country (see Bradley et al. 2000, 2007, 2016, 2022; Pérez-Consuegra and Vázquez‐Domínguez 2015, 2016; Matson et al. 2016).

### ﻿Species accounts

Based on our review, the species of *Peromyscus* that occur in Honduras are described below.

#### ﻿Rodentia Bowdich, 1821

**Myomorpha Brandt, 1855**

**Muroidea Illiger, 1811**

**Cricetidae Fischer, 1817**

**Neotominae Merriam, 1894**

***Peromyscus* Gloger, 1841**

***Peromyscusboylii* group**

##### ﻿ Peromyscus beatae

Taxon classificationAnimaliaRodentiaCricetidae

Thomas, 1903

4D528B8B-401F-50ED-958D-36C70425BBE7

###### Distribution.

Comayagua, Francisco Morazán, and Lempira departments (Fig. 2).

###### Ecoregions and elevation.

Central American montane, dry, and pine-oak forests (300–2850 m a.s.l.).

###### Comments.

The taxonomic classification of the *P.boylii* group has not undergone significant changes. However, Bradley et al. (2000) analyzed the taxonomy of the subspecies *P.boyliisacarensis* Dickey, 1928 using DNA sequences from the mitochondrial cytochrome *b* gene. As a result of this study, it is now considered as *P.beatae* (Bradley et al. 2000, 2017) (Table 1). Thus, specimens previously identified as *P.b.sacarensis* in Honduras should now be recognized as individuals of *P.beatae*. We verified the coordinates provided by Bradley et al. (2000), and the approximate coordinates corresponding to “2 mi NE El Hatillo” and “10 mi SE Tegucigalpa” and found them to be incorrectly attributed to the Olancho Department; these specimens belong to the department of Francisco Morazán. Cassola (2016) suggested the occurrence of *P.beatae* in other regions in western Honduras, but no tangible evidence corroborates this speculation.

#### ﻿*Peromyscusaztecus* group

##### ﻿ Peromyscus cordillerae

Taxon classificationAnimaliaRodentiaCricetidae

Dickey, 1928

45F44110-A56A-5FBE-81BC-CB55BEF4F62C

###### Distribution.

Lempira and La Paz departments (Fig. 3).

###### Ecoregions and elevation.

Central American dry and pine-oak forests (1129–1984 m a.s.l.).

###### Comments.

The individuals from Honduras that Goodwin (1942) had identified as *P.mexicanussaxatilis* Merriam, 1898 were considered to belong to *P.oaxacensis* Merriam, 1898, (*P.aztecus* group) by Musser (1969). Similarly, Musser (1969) indicated that the species cataloged as *P.hondurensis* Goodwin, 1941 by Goodwin (1942) also belonged to *P.oaxacensis*. Recently, Kilpatrick et al. (2021) treated *P.oaxacensis* as a subspecies of *P.aztecus* (Saussure, 1860) restricted to Mexico. Additionally, *P.cordillerae* refers to all members of the *P.aztecus* species group within the southeast of the Isthmus of Tehuantepec, and two subspecies have been provisionally proposed: one from the Cahuatique locality, as *P.cordilleraecordillerae*, and the other, *P.cordilleraehondurensis* for western Honduras (Table 2). Therefore, we should treat Honduran specimens referenced as *P.oaxacensis*, *P.aztecus*, *P.aztecusoaxacensis*, and *P.hondurensis* as pertaining to *P.c.hondurensis* (Musser 1969; Carleton 1979; Hall 1981; Kilpatrick et al. 2021). This group requires additional study in eastern Honduras and confirmation of its presence in other regions of the country. For example, Matson et al. (2016) identified *P.oaxacensis* (Table 2) based on its external morphology in the Sierra de Agalta National Park, situated in eastern Honduras, in the department of Olancho. This area is not part of the potential distribution proposed by Kilpatrick et al. (2021) which included the departments Choluteca, Comayagua, Copán, El Paraíso, Intibucá, Francisco Morazán, Ocotepeque, Santa Barbara, and Valle. Hence, it is crucial to verify these specimens and historical records before making any taxonomic reassignment.

#### ﻿*Peromyscusmexicanus* group

##### ﻿ Peromyscus nicaraguae

Taxon classificationAnimaliaRodentiaCricetidae

J. A. Allen (1908)

8E5F0BDD-5B67-547D-A3BA-CAFE21070DDC

###### Distribution.

Colón, Comayagua, Francisco Morazán, and Olancho departments (Fig. 4).

###### Ecoregions and elevation.

Central American montane, Atlantic moist forests, and pine-oak forests (1261–2030 m a.s.l.).

###### Comments.

*Peromyscusnicaraguae*, originally considered a distinct species, was later placed under *P.mexicanussaxatilis*. In a subsequent review, Musser (1969) reexamined the collections presented by Goodwin in 1942 in Honduras and determined that individuals previously identified as *P.guatemalensistropicalis* Goodwin, 1932 should be reclassified as *P.m.saxatilis*, both taxa being part of the *P.mexicanus* group. A recent taxonomic assessment conducted by Pérez-Consuegra and Vázquez-Domínguez (2015) significantly revised the *P.mexicanus* species group. Three junior synonyms were elevated to independent species status: *P.tropicalis* (formerly *P.g.tropicalis*), *P.nicaraguae* (previously *P.mexicanusnicaraguae*), and *P.salvadorensis* (Dickey 1928) (formerly *P.mexicanussalvadorensis*) (Table 3), these changes were based on the synonymy of *P.m.saxatilis* provided by Trujano-Álvarez and Álvarez-Castañeda (2010). Bradley et al. (2016) reported the northernmost locality in Honduras for this species in Capiro and Calentura National Park, without providing coordinates; therefore, it was approximated. However, the elevations in this area range from 600–1200 m a.s.l., suggesting that the species may occur at lower elevations in Honduras.

##### ﻿ Peromyscus salvadorensis

Taxon classificationAnimaliaRodentiaCricetidae

(Dickey 1928)

ED4C2F34-D016-5DA9-ABE7-04C50D022A0C

###### Distribution.

Lempira Department (Fig. 5).

###### Ecoregions and elevation.

Central American montane forests (1430–2870 m a.s.l.).

###### Comments.

Another species within the *P.mexicanus* group in Honduras is *P.salvadorensis* (Pérez-Consuegra and Vázquez-Domínguez 2015, 2016). Recent research indicates the presence of two lineages in the country: “lineage M” and “lineage L”, both displaying cryptic morphometric characteristics indicating a possible undescribed species (Table 3; Pérez-Consuegra and Vázquez-Domínguez 2015). According to Pérez-Consuegra and Vásquez-Domínguez (2016), however, both lineages inhabit mid to high elevations. Nevertheless, the specimens from lineages M and L reported in Pérez-Consuegra and Vásquez-Domínguez (2016), originate exclusively from the Celaque National Park in western Honduras.

*P.nicaraguae* and *P.salvadorensis* may be cryptic within their distribution ranges (Pérez-Consuegra and Vásquez-Domínguez 2016); however, these species are currently considered allopatric because *P.salvadorensis* has only been confirmed in western Honduras, and *P.nicaraguae* is documented in the central-eastern region of the country.

##### ﻿ Peromyscus stirtoni

Taxon classificationAnimaliaRodentiaCricetidae

Dickey, 1928

E4E4ABA2-CBC3-5974-8A58-E99D9186C9B5

###### Distribution.

Choluteca, Francisco Morazán, El Paraíso, and Valle departments (Fig. 6).

###### Ecoregions and elevation.

Central American dry and pine-oak forests (200–900 m a.s.l.).

###### Comments.

*P.stirtoni* was initially assigned to the *P.mexicanus* group (Hall and Kelson 1959). However, this placement was questioned and considered provisional by Carleton (1989). Subsequent mtDNA studies conducted by Bradley et al. (2007) and Ordóñez-Garza et al. (2010) suggested that *P.stirtoni* should be placed within the *P.megalops* species group. León-Tapia et al. (2022) reported that *P.stirtoni* forms a well-supported monophyletic lineage, but it is important to note its close relationship with lowland species, including the *P.megalops* group. In contrast, Bradley et al. (2016) considered *P.stirtoni* to be in the *P.mexicanus* group. Timm (2016) suggested that *P.stirtoni* might occur in La Paz, southern Comayagua, Lempira, Ocotepeque, and Intibucá departments, but this remains to be confirmed.

## ﻿Discussion

The *Peromyscus* genus in Honduras includes five recognized species: *P.beatae*, *P.cordillerae*, *P.nicaraguae*, *P.salvadorensis*, and *P.stirtoni*. However, this count might underestimate the actual diversity, and there could be additional *Peromyscus* species in Honduras. For example, there are potentially isolated populations of *P.nicaraguae* in northern and eastern Honduras (Pérez-Consuegra and Vázquez-Domínguez 2015, 2016; Matson et al. 2016). Similarly, the individuals identified by Matson et al. (2016) as “*P.oaxacensis*”, (currently *P.cordillerae*) in eastern Honduras deposited at the CMNH (Suppl. material 1) could yield additional species. Although the specimens from this museum are undergoing genetic studies (e.g., Pérez-Consuegra and Vázquez-Domínguez 2015, 2016), the review of the individuals identified as *P.oaxacensis* is of utmost importance as morphological and ecological evidence is required to confirm the distribution of *P.cordillerae* in Honduras (Kilpatrick et al. 2021).

The confirmed species count for the country might reflect the limited studies conducted on the genus in Honduras, primarily relying on specimens from museum collectors’ or curators’ identifications within historical collections. The uncertainty regarding the identity of Honduran *Peromyscus* individuals underscores the importance of validating historical specimens in the respective collections (Turcios-Casco et al. 2024), such as the ones of *Peromyscus* at the AMNH. This museum houses the largest collection of *Peromyscus* specimens (270) from Honduras and is comprised of specimens collected by one prominent mammalian collector for Honduras, C. F. Underwood, dating back to 1937. In contrast, the second-largest collection is deposited at the CMNH, has been used to demonstrate the presence of the genus in the country (Matson et al. 2016) and for taxonomic studies, such as those within the *P.mexicanus* and *P.boylii* groups (see Bradley et al. 2000; Pérez-Consuegra and Vázquez-Domínguez 2015, 2016; Bradley et al. 2016, 2017). Remarkably, there are regions in Honduras where the only known specimens are historical ones. For instance, the sole known specimens of *Peromyscus* from the La Paz Department were collected by C. F. Underwood in the 1930s (Goodwin 1942). Certain isolated specimens are regarded as genetic taxonomic units by some researchers, while others classify them as distinct forms. For instance, Kilpatrick et al. (2021) mentioned that the specimen TTU 83698 (TK 101037) labeled as *P.mexicanus* from La Tigra National Park (Francisco Morazán) was identified as *P.a.oaxacensis* in GenBank. However, the molecular analyses in Kilpatrick et al. (2021) suggest that the sequence of TK 101037 from Honduras aligns more closely with *P.nudipes* J.A. Allen, 1891 (sequence accession number FJ214675); and they proposed the possibility of contamination of this sequence with another taxon. We found no evidence confirming the presence of *P.nudipes* in Honduras. It is possible that similar cases may involve incorrect identification, however, we cannot confirm this possibility because we have not conducted reviews of specimens in scientific collections. On the other hand, *P.gymnotis* Thomas, 1894 is considered as potentially distributed in Honduras according to The International Union for the Conservation of Nature–IUCN (Vázquez and Reid 2016). In addition, Pérez-Consuegra and Vázquez Domínguez (2015) mentioned that *P.salvadorensis* (resurrected and elevated from synonymy with *P.m.saxatilis*) has been confused in the past with *P.gymnotis*. Hence, we propose a comprehensive examination of specimens from western and southwestern Honduras, where the species is anticipated to occur. Additionally, further collections from this region are essential to validate the presence of *P.gymnotis*.

From a conservation perspective, it is crucial to consider taxonomic checklists or reviews, as the work presented herein, because ignoring them can result in inaccurate conservation assessments. For instance, *P.gymnotis* is categorized as Data Deficient (DD) in the Red List of Honduras (WCS 2021). Unfortunately, *P.gymnotis* in Honduras has not been confirmed yet. Regrettably, in some cases, national classifications depend on extrapolating data, such as species expected to inhabit a particular area based on geographic proximity or limited available information, which can be misleading (e.g., Reid 2009). Similarly, *P.beatae* and *P.stirtoni* are categorized as DD in the Honduran Red List (WCS 2021); even though their presence has been confirmed additional information regarding their distribution, ecology, and natural history remains unknown. This classification in the Honduran Red List (WCS 2021) emphasizes the importance of conducting updated studies to accurately determine their true threat level and to propose and implement effective management measures for protecting their populations at the national level. For example, *P.mexicanus* sensu lato could be used to evaluate the effectiveness of protected area management (Cobo-Simón et al. 2018). However, it is essential to consider that in this region, three species of the *P.mexicanus* group (Pérez-Consuegra and Vázquez-Domínguez 2016; Bradley et al. 2016) and four species of the genus (*P.beatae*, *P.cordillerae*, *P.nicaraguae*, and *P.stirtoni*) could potentially coexist (see Figs 2–4, 6); all *Peromyscus* species for Honduras share significant similarities in their external morphology (Musser 1969; Hall 1981; Carleton 1989; Pérez-Consuegra and Vázquez-Domínguez 2015, 2016; León-Tapia et al. 2022).

The *P.mexicanus* group is known for exhibiting allopatric distributions with respect to its congeners, leading to cryptic speciation with conservative morphology (Ornelas et al. 2013; Pérez-Consuegra and Vásquez-Domínguez (2016)). This makes precise identification a challenge for research in the country (e.g., Cobo-Simón et al. 2018; Hoskins et al. 2018). Conservative morphology has also been mentioned for other groups of *Peromyscus* occurring in Honduras, such as *P.boylii* (Baird, 1855) and *P.aztecus* (Schmidly et al. 1988; Bradley et al. 2004, 2017), so elucidating both external and cranial morphological differences of *Peromyscus* should be a top priority. This is especially important because geographic variation complicates identification (Hall 1981). Previous studies must be complemented with geographic distribution, ecology, and the use of modern molecular techniques, such as mitochondrial DNA sequencing and phylogenetic analysis. Bradley et al. (2016) have employed these techniques to clarify the systematics of *P.nudipes* and *P.nicaraguae*, including specimens previously assigned to *P.nudipeshesperus* Harris, 1940 and *P.nudipesorientalis* Goodwin, 1938 from Costa Rica, Honduras, Nicaragua, and Panamá. This integration of molecular data with information from genetic databases like GenBank facilitates the assessment of genetic variability within *Peromyscus*, paving the way for future research in this field (Bradley et al. 2016).

This review serves as a crucial foundation for future investigations, highlighting the need for a comprehensive understanding of species diversity and the taxonomy of *Peromyscus* in the region. By addressing taxonomic uncertainties and consolidating available data, this study paves the way for more accurate conservation assessments and informed management strategies. It also sets a precedent for ongoing research efforts aimed at elucidating the biodiversity and evolutionary history of *Peromyscus* species within the Honduran context.

## Supplementary Material

XML Treatment for Peromyscus beatae

XML Treatment for Peromyscus cordillerae

XML Treatment for Peromyscus nicaraguae

XML Treatment for Peromyscus salvadorensis

XML Treatment for Peromyscus stirtoni

## ﻿Additional information

### Author ORCIDs

Celeste M. López https://orcid.org/0000-0002-7002-7511

Manfredo A. Turcios-Casco https://orcid.org/0000-0002-3198-3834

Eric van den Berghe https://orcid.org/0000-0001-7566-0415

Nicté Ordóñez-Garza https://orcid.org/0000-0002-7732-7824

Martin R. Alvarez https://orcid.org/0000-0001-6908-8547

### Data availability

All of the data that support the findings of this study are available in the main text or Supplementary Information.

## Supplementary materials

Supplementary material 1 Verified records of *Peromyscus* in Honduras Data type xlsx Explanation note Verified occurrence list of *Peromyscus* species in Honduras, including the museums where they are deposited, catalog numbers, as well as a description for each record including the department, municipality, and locality with geographical coordinates in decimal format and WGS-84 Datum. Please refer to observations for detailed remarks on each specimen. https://binary.pensoft.net/file/1180693This dataset is made available under the Open Database License (http://opendatacommons.org/licenses/odbl/1.0/). The Open Database License (ODbL) is a license agreement intended to allow users to freely share, modify, and use this Dataset while maintaining this same freedom for others, provided that the original source and author(s) are credited.Celeste M. López, Manfredo A. Turcios-Casco, Eric van den Berghe, Nicté Ordóñez-Garza, Martin R. Alvarez
